# The prevalence of primary headache disorders in Saudi Arabia: a cross-sectional population-based study

**DOI:** 10.1186/s10194-020-1081-1

**Published:** 2020-02-07

**Authors:** Mohammed Al Jumah, Ali M. Al Khathaami, Suleman Kojan, Mohamed Hussain, Hallie Thomas, Timothy J. Steiner

**Affiliations:** 1 King Abdullah International Medical Research Centre, Riyadh, Saudi Arabia; 2 King Saud Bin Abdulaziz University for Health Sciences, Riyadh, Saudi Arabia; 3 Department of Neurosciences, King Fahad Medical City, MOH, Riyadh, Saudi Arabia; 4 King Abdulaziz Medical City, Riyadh, Saudi Arabia; 5 Department of Neuromedicine and Movement Science, Faculty of Medicine and Health Sciences, NTNU Norwegian University of Science and Technology, Trondheim, Norway; 6 Division of Brain Sciences, Imperial College London, London, UK

**Keywords:** Epidemiology, Prevalence, Headache, Migraine, Tension-type headache, Medication-overuse headache, Saudi Arabia, Eastern Mediterranean Region, Global Campaign against Headache

## Abstract

**Background:**

The large geographical gaps in our knowledge of the prevalence and burden of headache disorders include most of Eastern Mediterranean Region (EMR). Following a nationwide population-based study in Pakistan, we present here a similar study from Kingdom of Saudi Arabia (KSA). Both were conducted as projects within the Global Campaign against Headache The two purposes of this study were to inform national health policy and contribute to global knowledge of headache disorders.

**Methods:**

We surveyed Arabic-speaking adults aged 18–65 years in all 13 regions of KSA. While previous Global Campaign studies have engaged participants by calling at randomly selected households, the culture of KSA made this unacceptable. Participants were, instead, contacted by cell-phone (since cell-phone coverage exceeded 100% in KSA), using random-digit dialling. Trained interviewers used a culturally adapted version of the Headache-Attributed Restriction, Disability, Social Handicap and Impaired Participation (HARDSHIP) questionnaire, with diagnostic enquiry based on ICHD-II. We estimated 1-year prevalences of the headache disorders of public-health importance (migraine, tension-type headache [TTH] and probable medication-overuse headache [pMOH]) and examined their associations with demographic variables.

**Results:**

A total of 2316 participants (mean age of 32.2 ± 10.7 years; 62.3% male; 37.7% female) were included (participation proportion 86.5%). Gender and age distributions imperfectly matched those of the national population, requiring adjustments for these to prevalence estimates. Observed 1-year prevalence of all headache was 77.2%, reducing to 65.8% when adjusted. For headache types, adjusted 1-year prevalences were migraine 25.0%, TTH 34.1%, pMOH 2.0% and other headache on ≥15 days/month 2.3%. Adjusted 1-day prevalence of any headache was 11.5%. Migraine and pMOH were associated with female gender (ORs: 1.7 and 4.7; *p* < 0.0001). Migraine was negatively associated with age > 45 years (OR: 0.4; *p* = 0.0143) while pMOH was most prevalent in those aged 46–55 years (OR: 2.7; *p* = 0.0415). TTH reportedly became more common with increasing level of education.

**Conclusion:**

Prevalences of migraine and TTH in KSA are considerably higher than global averages (which may be underestimated), and not very different from those in Pakistan. There is more pMOH in KSA than in Pakistan, reflecting, probably, its higher-income status and greater urbanisation (facilitating access to medication).

## Introduction

Over a decade ago, a systematic review of the published literature found that headache disorders affected almost half the world’s population [1]. Tension-type headache (TTH) and migraine, both primary headache disorders [2], were the major contributors to headache prevalence. Later, in various iterations of the Global Burden of Disease (GBD) study, they were shown to be the second and third most common disorders in the world [3] and of clear public-health importance [3–8]. Medication-overuse headache (MOH), not itself a primary headache disorder but in almost all cases a sequela due to mismanagement of either migraine or TTH, is among the group of disorders characterized by headache on ≥15 days/month [2]. Consequently it, too, is an important contributor to public ill health.

The same review showed large geographical gaps in our knowledge [1]. *Lifting The Burden* (LTB), conducting the Global Campaign against Headache [9–12], has since been supporting a series of population-based studies to fill these gaps [13–20]. Since there were, in 2007, no data on headache from the whole of the Eastern Mediterranean Region (EMR) [1], these studies have included Pakistan [20], an EMR country with the sixth largest population in the world. Pakistan, however, characterised by economic and political instability and, in parts, by geographical inaccessibility, cannot be considered typical of the Region, which includes the Arab Gulf countries and many in North Africa, from which Pakistan differs ethnically and culturally. Accordingly, we report here a second Global Campaign study from EMR, with prevalence findings of a nationwide cross-sectional population-based survey in the Kingdom of Saudi Arabia (KSA). This country, although second behind Algeria in the Arab world, constitutes the bulk of the Arabian Peninsula and is the largest Arab state by land in Western Asia area (2.15 million km^2^ [21]). Its total population according to the last official census in 2010 was a little over 27 million [22], but later estimates, for 2015–17, vary between 28.6 million, with well over 30% non-Saudi immigrants creating some uncertainty [22, 23], and about 32 million [21].

All Global Campaign studies so far have followed standardized methodology developed by LTB [24]. In KSA, cultural considerations made methodological adaptations necessary. In particular, while interviewers in all previous LTB-supported studies have engaged participants by calling unannounced at randomly selected households [24], this would not have been acceptable in KSA. Furthermore, however it was achieved, helpful engagement with female participants required female interviewers.

As has been the case for all Global Campaign studies, the two purposes here were to provide evidence for national health policy and to contribute to GBD.

## Methods

### Ethics

The Ethics Review Board of King Abdullah International Medical Research Centre approved the study protocol. All participants were informed about the nature and purpose of the survey and gave their consent to taking part.

Data-protection legislation was complied with. Personal data were anonymised during analysis and dissemination.

### Study design

This was a cross-sectional questionnaire-based survey of adults aged 18–65 years. It was designed in accordance with published guidelines [24], albeit with culturally mandated adaptations. We randomly selected and engaged with participants by cell-phone, using random digit-dialling (RDD).

### Setting and population

We sampled from all 13 regions of Saudi Arabia, according to the population size of each established by the 2010 national census [22].

### Sampling method

We aimed for a total sample size of 2000, in line with published guidelines [24]. For planning purposes we anticipated a 20% non-participation proportion and a requirement therefore to contact a minimum of 2400 potential respondents (N), drawn from each region in proportion to its population according to the census [22].

A total of 10,000 Saudi cell-phone numbers were generated by web-based random-numbers generator software (Research Randomizer) [25]. Almost 6000 of these turned out to be invalid, out of reach, out of service or unanswered despite two calls. The 4000 others became the means of contact and engagement with potential participants, and were called until recruitment was sufficient. These numbers might be in any region; therefore, having determined the number (n) required in each region, once we reached n we ceased enrolling from that region.

Any Arabic-speaking person aged 18–65 years who answered the telephone was a potential participant. The survey therefore excluded non-Arabic-speaking immigrants but was not limited to Saudi nationals. Any younger person who answered was asked to pass the phone to an older family member, or the call was terminated. Numbers not answered when first dialled were called a second time.

### Selection and training of interviewers

The interviewers were research coordinators with nursing or allied health backgrounds, trained in questionnaire administration and with an understanding of the study and its purposes.

### Data collection

Each eligible person who agreed upon first contact to participate was included in the survey and, whenever willing, interviewed immediately through the cell-phone call. Those who preferred to be interviewed at another time were phoned again by appointment, when expected to be available. We respected cultural considerations by allowing all female participants the opportunity to be questioned by female interviewers, although many declined this offer.

Interviews followed the Headache-Attributed Restriction, Disability, Social Handicap and Impaired Participation (HARDSHIP) structured questionnaire [26], culturally modified and translated into Arabic according to LTB’s translation protocol for lay documents [27]. The questionnaire had four parts: a) personal and demographic enquiry (including gender, age, marital status, educational level, employment and income from all sources), and b) headache screening questions, which were addressed to all participants; these were followed in those screening positively by c) diagnostic questions based on ICHD-II [28] and d) enquiry into burden. The screening question for headache was: “Have you had a headache during the last year?” Participants who answered “no” were classified as headache-free; those who answered “yes” were asked if all their headaches were of one or more types and, if more than one, to focus in the subsequent questions on the one that was subjectively the most bothersome. The point prevalence of headache was estimated by asking: “Did you have a headache yesterday?”

Data were collected electronically by interviewers directly into the study database, held securely in the data management unit at King Abdullah International Medical Research Centre.

### Diagnosis

Interviewers did not make diagnoses: these were derived algorithmically during analysis [26]. We first separated participants reporting headache on ≥15 days/month, describing these as a separate group because they cannot be fully diagnosed by questionnaire. Those among this group who also reported regular use of headache medication on > 3 days/week were considered to have probable MOH (pMOH). To all others, the algorithm applied ICHD-II criteria in the recommended order: migraine, TTH, probable migraine, probable TTH [26, 28]. Again as recommended, cases of migraine and probable migraine, and of TTH and probable TTH, were combined for prevalence estimation and further analyses [24, 26]. Remaining cases were unclassified.

### Quality control

Throughout the period of data collection, for fraud-detection purposes, participants were randomly selected and called again, at the rate of approximately 2/week. The first purpose was to establish that the phone number was valid. The second, to establish that the reported interview took place, was achieved by asking the respondent two questions: (1) “Did anyone call you from the King Abdullah International Medical Research Centre?”, and (2) “What was the call about?” No concerns were generated, and the data were not, therefore, systematically recorded.

### Statistics and analysis

We used descriptive summaries for sociodemographic and clinical characteristics. We recorded gender as male or female and analysed these as proportions (%). We recorded age in years, calculating mean ± standard deviation (SD), but analysed it as a categorical variable (18–25, 26–35, 36–45, 46–55, 56–65 years). We classified marital status as single (never married), married, or widowed/divorced; educational level as none, school, or university; employment status as housewife (no other employment), full-time employed/self-employed, student, or otherwise not working (including not currently working but seeking work, and retired: these groups combined because of small numbers). Income data were not analysed for reasons stated later (see Results).

We estimated prevalences as proportions (%) with 95% confidence intervals (CIs), adjusting observed values for gender and age by separately estimating each prevalence in all 10 gender-age groupings and weighting the mean according to the population proportions of these groupings [22]. We used chi-squared and Fisher’s exact tests in comparisons of proportions and distributions. In bivariate analyses for associations between headache types and sociodemographic variables, we calculated odds ratios (ORs) with 95% CIs. We regarded *p* < 0.05 as significant.

Analyses were performed using SPSS/PC version 20.0 software package for statistical analysis (SPSS, INC, Chicago, IL) and Excel Professional Plus 2010 Version 14.0.7166.5000. All available data were analysed, with no imputations for missing data.

## Results

From approximately 4000 phone numbers called and answered, 2800 identified eligible respondents; the remainder were either terminated by the interviewer because the respondent was ineligible or from a region already fulfilled, or declined by the respondent before eligibility could be established. In accordance with guidelines, these were not regarded as non-participants [24]. Of the 2800 initially judged eligible, 2421 participated in the interview (participation proportion 86.5%). However, 105 were later excluded, being outside the intended age range of 18–65 years; therefore, 2316 (final N) were analysed.

The sociodemographic characteristics of participants (Table 1) imperfectly matched those of the national population, with important discrepancies in gender and age. Males in the sample (62.3%) considerably outnumbered females (37.7%). To a large extent this reflected the gender distribution of the population, distorted by > 30% of immigrant workers who are mostly male [20, 21], but the sample was significantly different from the population according to 2010 census data [20] (Table 1). Mean age of the sample was 32.2 ± 10.7 years, with progressive under-representation of age groups above 35 years (Table 1). Although age distribution within the sample also reflected distortions within the population [20, 21], differences were again significant overall. Since gender and age both influence headache prevalence, we adjusted observed values for these. Participants were relatively well-educated, and people with university education were over-represented in the sample (Table 1). Most participants were married, but those who had never done so were nonetheless slightly over-represented in the sample (Table 1). Occupational categories in the sample differed from national proportions by no more than 2–4%. Three quarters of the sample were Saudi nationals, with non-Saudis therefore under-represented (Table 1). Geographic distribution was assumed to reflect the national population spread because the sampling method ensured this.

**Table 1:** Sociodemographic characteristics of study participants (*N* = 2316) and of the national population

Characteristic	Sample *n* (%)	National population^a^ %	Chi-squared *p*
Gender			
male	1442 (62.3)	60.6	30.23
female	874 (37.7)	39.4	< 0.0001
Age (years)			
18–25	769 (33.2)	16.6	
26–35	818 (35.3)	29.1	233.8
36–45	448 (19.3)	29.3	< 0.0001
46–55	199 (8.6)	17.0	
56–65	82 (3.5)	7.9	
Education			
none	205 (8.9)	11.7	724.8
school	1212 (52.3)	70.8	< 0.0001
university	897 (38.8)	17.5	
Marital status			
never married	867 (37.5)	33.3	30.89
married	1381 (59.7)	62.0	< 0.0001
widowed/divorced	66 (2.8)	4.7	
Occupation			
employed or self-employed (*n*=)	1199 (51.9)	53.6	
housewife	567 (24.5)	22.6	68.56
student	348 (15.1)	18.6	< 0.0001
other (including retired)	196 (8.5)	5.2	
Nationality			
Saudi	1730 (74.7)	66.0	78.1
non-Saudi	586 (25.3)	34.0	< 0.0001

^a^from national census 2010 [20]

### Prevalence

In total, 1789 participants (77.2% [95% CI: 75.5–79.0%]) reported headache in the preceding year. Gender- and age-adjusted 1-year prevalence was 65.8% [63.9–67.7%]. Table 2 shows observed and adjusted 1–year prevalences of each headache type, the latter reflecting the divergence of sample demographics from those of the national population. TTH was the most common headache type overall (observed 42.9% [definite 36.3%, probable 6.6%; adjusted 34.1%], compared with migraine 28.7% [definite 11.5%, probable 17.2%]; adjusted 25.0%), and in both genders. All causes of headache on ≥15 days/month were 4.3% (adjusted), of which almost half (2.0% adjusted) were pMOH. There were only 41 cases (1.8%) of unclassified headache.

**Table 2:** Observed 1-year prevalence (%) by headache type, gender and age, observed 1-day prevalence of any headache (headache yesterday), and adjusted values for gender and age

	Migraine (*n* = 663)	Tension-type headache (*n* = 994)	Probable MOH (*n* = 41)	Other headache on ≥ 15d/m (*n* = 48)	Any headache yesterday (*n* = 254)
All (*N* = 2316)	28.7 [26.9–30.6]	42.9 [40.9–45.0]	1.8 [1.3–2.4]	2.1 [1.5–2.7]	11.0 [9.7–12.3]
Gender					
male (*n* = 1442)	24.3 [22.1–26.6]	45.3 [42.7–47.9]	0.8 [0.4–1.4]	1.4 [0.8–2.1]	8.8 [7.4–10.4]
female (*n* = 874)	36.0 [32.9–39.3]	39.0 [35.8–42.3]	3.4 [2.4–4.9]	3.2 [2.3–4.6]	14.5 [12.3–17.0]
*p* (male vs female)^a^	< 0.0001	0.0032	< 0.0001	0.0039	< 0.0001
Age (yr)					
18–25 (*n* = 769)	31.1 [27.8–34.5]	41.4 [37.8–44.9]	1.3 [0.6–2.4]	2.1 [1.2–3.4]	10.3 (8.2–12.6]
26–35 (*n* = 818)	28.0 [24.9–31.2]	45.7 [42.3–49.2]	1.6 [0.8–2.7]	1.7 [0.9–2.9]	10.6 [8.6–13.0]
36–45 (*n* = 448)	29.5 [25.3–33.9]	42.0 [37.3–46.7]	2.2 [1.1–4.1]	2.7 [1.4–4.6]	11.6 [8.8–14.9]
46–55 (*n* = 199)	24.6 [18.8–31.2]	39.7 [32.8–46.9]	3.5 [1.4–7.1]	2.5 [0.8–5.8]	13.1 [8.7–18.6]
56–65 (*n* = 82)	19.5 [11.6–29.7]	42.7 [31.8–54.1]	1.2 [0.0–6.6]	1.2 [0.0–6.6]	12.2 [6.0–21.3]
Adjusted for gender and age^b^	25.0 [23.2–26.8]	34.1 [32.2–36.0]	2.0%	2.3%	11.5%

*MOH* medication-overuse headache, *d/m* days/month; prevalence values are % [95% confidence interval]; ^a^Fisher’s exact test, 2-tailed; ^b^adjusted by correcting for gender and age distribution according to national census 2010 [20]

Headache yesterday [HY] was reported by 254 participants (11.0% [9.7–12.2%]). Adjusted 1-day prevalence of any headache was 11.5% (Table 2).

### Associations

All headache types except TTH were significantly more common in females (Table 2), migraine by about 3:2, pMOH by about 4:1 and other headache on ≥15 days/month by about 2:1. HY was reported nearly twice as commonly by females.

Migraine was most common in those aged 18–25 years, declining only slightly until 36–45 years, and then more steeply (Table 2). For TTH, the age-relationship was rather flat across all ages, with only a small peak among those aged 26–35 years. For pMOH, with small numbers and wide CIs, reported prevalence increased steadily up to 46–55 years and then declined sharply. Other headache on ≥15 days/month also declined after 46–55 years, again with small numbers. HY was reported more as age increased up to 46–55 years, but this was a non-significant trend. ORs for these associations are in Table 3.

**Table 3:** Logistic regression analysis of associations of sociodemographic variables with the principal headache types

	Migraine		Tension-type headache		Probable MOH	
	OR [95% CI]	*p*	OR [95% CI]	*p*	OR [95% CI]	*p*
Gender						
male (*n* = 1442)	reference	–	reference	–	reference	–
female (*n* = 874)	**1.7 [1.4–2.1]**	**< 0.0001**	0.9 [0.7–1.0]	0.1237	**4.7 [2.3–9.4]**	**< 0.0001**
Age (yr)						
18–25 (*n* = 769)	reference	–	reference	–	reference	–
26–35 (*n* = 818)	0.9 [0.7–1.1]	0.2419	1.1 [0.9–1.4]	0.2244	1.4 [0.6–3.2]	0.4277
36–45 (*n* = 448)	0.9 [0.7–1.2]	0.4621	0.9 [0.7–1.2]	0.5559	1.7 [0.7–4.2]	0.2231
46–55 (*n* = 199)	0.7 [0.5–1.0]	0.0626	0.9 [0.6–1.3]	0.5770	**2.7 [1.0–7.4]**	**0.0415**
56–65 (*n* = 82)	**0.4 [0.2–0.8]**	**0.0143**	0.9 [0.5–1.5]	0.6904	0.9 [0.1–7.4]	0.9509
Education						
none (*n* = 205)	1.0 [0.7–1.4]	0.8549	**0.3 [0.2–0.5]**	**< 0.0001**	1.8 [0.6–4.8]	0.2731
school (n-1212)	reference	–	reference	–	reference	–
university (*n* = 897)	1.1 [0.9–1.4]	0.2720	**1.3 [1.0–1.5]**	**0.0142**	1.5 [0.8–2.9]	0.2129
Employment						
employed or self-employed (*n* = 1199)	reference	–	reference	–	reference	–
housewife (*n* = 567)	**1.5 [1.2–1.8]**	**0.0012**	0.8 [0.7–1.0]	0.0735	**2.4 [1.2–4.9]**	**0.0127**
student, seeking work or retired (n-544)	1.2 [0.9–1.5]	0.1504	0.9 [0.7–1.1]	0.2069	1.3 [0.6–3.1]	0.504

*MOH* medication-overuse headache, *OR* odds ratio, *CI* confidence interval; significant differences are emboldened

Logistic regression analysis (Table 3) confirmed the positive associations of migraine and pMOH with female gender and negative association of migraine with age > 45 years, and demonstrated that pMOH was most prevalent in those aged 46–55 years. Analyses of associations with socioeconomic indicators (education and employment) revealed nothing unexpected (Table 3): gender associations were reflected in associations with the (obviously female) occupation of housewife. The only other finding of note was that TTH reportedly increased in prevalence with level of education. Income data were not analysed because they were reported by only 782 participants (33.8%), and probably highly biased.

## Discussion

This study in KSA, the second population-based study in EMR using Global Campaign methodology, has found headache disorders to be very common in this country. Almost two thirds of adults (65.8%) are affected, reporting at least one episode in the preceding year. We estimated 1-year prevalences, gender- and age-adjusted, of 25.0% for migraine and 34.1% for TTH. Headache on ≥15 days/month affects 4.3%, and 11.5% have headache on any 1 day (generalizing from HY, on the assumption that there was nothing special about “yesterday”).

In questioning the reliability of these estimates, we draw attention to two important study limitations.

The first was sampling, and engagement with participants, by cell-phone, culturally necessitated but a suboptimal method [24]. In KSA, use of cell-phones is almost universal: in 2012, according to a United Nations Conference on Trade and Development (UNCTAD) report, KSA led the world, with a ratio – predicted to continue rising – of 1.88 cell-phones per head of population [29]. A ratio of > 1 is itself problematic, since holders of multiple phones have an enhanced chance (multiplied by the number of phones) of being selected. Predictably, sampling by cell-phone would lead to under-selection of females, older people, non-Saudis (who might have non-Saudi phone numbers) and those in poverty. This appears to have happened, at least with respect to the first groups, but not to such an extent that adjustment could not achieve statistical correction. While RDD is an accepted sampling method, it is more likely than cold-calling at households to allow the introduction of participation bias: a phone call is readily terminated – more than might be the case by a respondent engaged face-to-face and at home by an interviewer [24]. This bias might be against those with no interest in the survey (those without headache), or those who might consider themselves too busy (professional classes), but the participation proportion of 86.5% suggests it was not a highly influential factor. An important safeguard was that female interviewers were always available to question female participants who expressed a preference for this.

The second limitation was largely a consequence of the first. Validation of the diagnostic questions requires second interviews by a headache expert of a subset of participants. This was difficult to arrange when the initial engagement was by cell-phone, and risked selection of a highly biased sub-sample – those with particularly troublesome headache. It was attempted, but abandoned, since the questionnaire had a record of successful use in many countries and cultures [14–20] and had been validated in four of these [14–16, 20, 26].

With these issues in mind, what did our study show? The global age-standardised 1-year prevalence estimates for migraine and TTH, from GBD2016 [6] (which has the most detailed headache analyses), are 14.4% and 26.1%. Our findings of 25.0% migraine prevalence and 34.1% for TTH are higher than these global means by, respectively, factors of 1.7 and 1.3. These, particularly the first, are large margins of difference. While headache to some extent affects almost two thirds of adults (65.8%) in KSA, one in every 23 has headache on more days than not. On any particular day, one in every nine has headache. This indicates a substantial amount of headache in the country, and of consequential ill health, with a strong association, except for TTH, with female gender.

While we might, speculatively, try to explain these high prevalences in terms of genetic or cultural factors specific to Saudi Arabia, it may be more relevant to question the global estimates of GBD, which still lack data from many parts of the world. GBD2016 also estimated case counts in KSA – by extrapolation, since no direct data were available – of 5.8 million migraine and 8.7 million TTH. On the basis that about two thirds (67%) of the total population of KSA are aged 18–65, or 21.3 million people [21, 23], there are, according to our estimate, 5.3 million people with migraine among these alone. Prevalence in the almost 10 million aged < 18 years is expected to be lower (we have no data about these yet), but nevertheless the GBD2016 estimate of 5.8 million in total looks low, as, correspondingly, does the estimate for TTH. Future iterations of GBD will be better informed.

Comparisons with the global estimates of GBD are supplemented, perhaps more informatively, by comparisons within the region. The only data for comparison from the Arab world are from a Kuwaiti study, performed after ours, with interviewers using the HARDSHIP questionnaire while calling at households [30] (in theory, a better methodology). But, despite a very large reported N (15,523), the participation proportion was only 53.3% – a potential source of serious bias. Prevalence of episodic migraine (presumably definite + probable) was 23.1% (95% CI 19.4–32.9%), which would not, on these values, differ significantly from our finding in KSA (but it has to be noted that the reported 95% CI implies a much smaller N than 15,523, for which the 95% CI would have been 22.5–23.8%). Prevalence of “chronic headache” (probably equivalent to our headache on ≥15 days/month) was 5.4% (no CI reported). Also problematic in the Kuwaiti study is that no adjustments in prevalence estimates appear to have been made for gender or age [30].

Otherwise, from all of EMR, there are published findings only from Pakistan, a Global Campaign study of 4223 participants (mean age 34.4 ± 11.0 years) sampling, as in Kuwait, by calling at households, but with a much higher participation proportion of 89.5% [20]. Despite the methodological differences from our study in KSA (but use of the same questionnaire [20]), we can point to broadly similar findings. In Pakistan the age- and gender-adjusted 1-year prevalence of migraine is 22.5% [95% CI: 21.2–23.8%]; in KSA, at 25.0 [23.2–26.8], it is somewhat higher (Fisher’s exact test: *p* = 0.0238). In both countries, migraine is more prevalent in females by a factor of 3:2 [20], whereas globally it is close to 2:1 [6]. Values for TTH are more different: in Pakistan 44.6% (43.1–46.1%), in KSA 34.1 (32.2–36.0%; *p* < 0.0001). However, TTH is more prevalent in males in both countries, in Pakistan by about 1.3:1, in KSA by 1.2:1. This unusual gender differential, seen also in Nepal [17], is possibly explained by male:female working ratios in mostly patriarchal countries: in Pakistan the burden of supporting the entire household falls almost solely on the male [31], whereas in KSA, the labour force is over 80% male [23] (even among Arab countries, KSA has one of the lowest levels of female participation in the national labour force). Our finding in KSA that TTH prevalence is positively associated with educational level suggests an influence of level of responsibility, but this is speculative.

The prevalence of pMOH in KSA probably also exceeds the global mean [32]. There is a large gender-related difference in pMOH between KSA and Pakistan: while this disorder affects only 0.8% of adults in Pakistan [20], and the same proportion of males in KSA, it is reported by 3.4% of females in KSA, making it predominantly a problem among women in this country. This disorder is typically more prevalent among females [32]. Migraine prevalence is the principal driver of MOH, since migraine is the antecedent headache in most cases, but access to medication is a key risk factor. Unlike Pakistan, KSA is a high-income country [33], but wealth is distributed very unequally. Whether this is a relevant factor is very uncertain; more likely is the higher urbanisation rate in KSA (> 83% [23], versus about 40% in Pakistan [34]). In Zambia, lower-middle-income like Pakistan, and with very similar urbanisation [35], urban dwelling was a major factor positively associated with pMOH prevalence.

Pakistan and KSA are very different geographically, ethnically, culturally, politically, economically and in urbanisation rates. There are differences also in terms of headache, possibly consequentially, but they are not large. Overall, Pakistan has rather more prevalent headache than KSA (76.6% and 65.8% respectively), but this is accounted for by TTH, the least burdensome of the headache types [6]. There is more pMOH in KSA, reflecting, probably, the higher-income status of KSA and its greater urbanisation, which both facilitate access to medication.

## Conclusion

Prevalences in KSA of migraine and TTH, and perhaps of pMOH, are considerably higher than global averages. The latter are almost certainly underestimated. Prevalences in KSA are not very different from those in Pakistan, also an EMR country but with many differences otherwise.

Information on the burden attributable to these disorders in KSA, needed to inform health policy and priority-setting, will be reported later.

## Data Availability

Full electronic data are held securely at King Abdullah International Medical Research Centre and the analytical subset at Norwegian University of Science and Technology (NTNU), Trondheim, Norway. When analyses are completed, the latter will be available on request for academic purposes.

## Abbreviations

CI: Confidence interval
d/m: days/month
EMR: Eastern Mediterranean Region
GBD: Global Burden of Disease
HARDSHIP: Headache-Attributed Restriction, Disability, Social Handicap and Impaired Participation questionnaire
ICHD: International Classification of Headache Disorders
KSA: Kingdom of Saudi Arabia
LTB: *Lifting The Burden*
MOH: Medication-overuse headache
OR: Odds ratio
pMOH: probable MOH
RDD: Random digit-dialling
SD: Standard deviation
TTH: Tension-type headache
